# FEM analysis of a new three-way drainage and pressure reduction system for road tunnels

**DOI:** 10.1038/s41598-023-37417-1

**Published:** 2023-07-05

**Authors:** Zhaolei Teng, Yuanming Liu, Shilong Mei, Yuhang Zhou, Guohua He, Yingxiao Li, Bitao Du

**Affiliations:** 1 College of Civil Engineering, Guizhou University, Guiyang, 550025 China; 2 Guizhou Provincial Key Laboratory of Rock and Soil Mechanics and Engineering Safety, Guiyang, 550025 China

**Keywords:** Civil engineering, Ecology

## Abstract

For water-rich areas, tunnel elevation arches under high water pressure often cause elevation arch cracking and leakage, bulging and other failures. When the drainage system is not designed properly, these failures occur more frequently, and conventional road tunnel drainage cannot effectively reduce the water pressure at the elevation arch. Therefore, this paper proposes a new concept of "three-way drainage". The three-way drainage system is based on a conventional drainage system with a new drainage inlet at the elevation arch. On this basis, a series of numerical simulation studies are conducted to verify the pressure-reducing performance of the three-way drainage system on the lining. After demonstration and analysis, the three-way drainage concept can not only effectively reduce the water pressure at the elevation arch of the tunnel but also have a significant effect on the overall drainage effect of the tunnel. The factors affecting the performance of the three-way drainage system are assessed by varying the model parameters. It is found that the hydraulic conduction coefficient of the surrounding rock and initial support, the number of reverse diversion holes in the elevation arch, the change in head height, and the change in secondary lining parameters all have a significant effect on the water pressure outside the tunnel.

## Introduction

A country's economic development is inevitably linked to the construction of roads, railroads and other infrastructure. China has made remarkable achievements in tunnel construction in recent years. For example, by the end of 2020, China's total road mileage reached 5,198,100 km, an increase of 185,600 km over 2019, and China's road tunnels reached a whopping 21,316 km and a total distance of 21,999,300 linear meters. During the construction of road tunnels, several failure incidents have occurred. Among these problems, the cracking of road tunnel linings caused by high water pressure has become a serious factor affecting tunnel safety and has also attracted industry attention^1,2^. Types of tunnel lining cracking failures include arch wall cracking, high-pressure water injection, and elevation arch bulging^3–6^. A bulging roadbed of a road tunnel will not only seriously affect the service life of the tunnel but also present a safety hazard for traffic. Even if an elevated arch bulge on a highway is not obvious, it can still cause a terrible traffic accident for vehicles driving at high speed^7,8^. A tunnel elevation arch bulge may not be solely due to high ground stress caused by high water pressure; if the flood season drainage in a tunnel is not timely, high water pressure concentrated in the elevation arch can leads to rock absorption and expansion, which can soften the rock and lead to a tunnel elevation arch bulge. Therefore, it is crucial to economically and effectively solve problems such as high water pressure causing damage to a tunnel lining structure. At present, there are many ways to reduce lining damage, among which an effective and efficient drainage system is currently the main way to reduce pressure on a tunnel^9^.

Currently, there are two main highway tunnel waterproofing and drainage strategies: a fully enclosed model that does not allow groundwater to flow into the tunnel and a drainage model that allows groundwater to flow into the tunnel. The fully enclosed model is often used for special locations such as natural environment protection areas and locations where there are important buildings on the ground that cannot be drained by the tunnel for a long time, causing subsidence. Generally, the strength requirements of the lining structure and the waterproof layer are high. Therefore, for nonnatural protected areas, drainage systems are generally used to reduce the external water pressure on the tunnel lining. Scholars at home and abroad are currently studying how high water pressure affects the structural stress characteristics of tunnel linings through a series of theoretical analyses, model tests, field tests and numerical simulations^10–15^. Theoretically, the spatial distribution of pore water pressure in urban tunnels in water-rich areas was studied based on Harr, and a water pressure equation for the seepage field was derived^16^. Complex variable analysis was used to analyze the stress distribution in elastic half-plane underwater tunnels^17^. A semianalytical approach to tunnel water inflow was proposed^18^. A structural form suitable for high water level tunnels and the structural form of a controlled drainage scheme were proposed, and the water pressure distribution in a tunnel lining was studied by theoretical analysis, indoor tests and field measurements^19^. An assessment framework based on a regionally coupled hydrological model was developed to study the effects of tunnel drainage on surrounding vegetation^20–23^. However, the current research results of tunnel drainage prevention involved conventional drainage methods. Although this has a significant effect on water pressure reduction outside the lining compared to full closure, it only has a significant pressure reduction effect on the pressure around the tunnel arch wall. On the material side, in-plane permeability tests of drained geotextile filters were conducted to assess their mechanism of hydraulic deterioration in tunnels^24,25^. An optimal lightweight foam mortar mixture for promoting tunnel drainage was examined using the composite lining method^26^. Waterproof and breathable materials were designed based on electrospun nanofibers^27^. In terms of structure, a new drainage structure containing convex hull drainage slabs was proposed by numerical simulation and indoor testing^28^. A tunnel drainage system was constructed by 3D printing technology, and a simulation test of drainage system blockage was performed^29^. Three waterproofing and drainage optimization schemes were proposed, where placing a central drain at the bottom of an invert had the greatest impact on reducing water pressure by 96%^9^. A bottom-up railroad tunnel drainage method with pressure reduction was proposed^30^. A new drainage network was proposed to solve the drainage problem of mountain tunnels crossing high LWP fracture zones^31^. They studied and analyzed the waterproof requirements and construction measures of different special tunnels in China^32–34^. The above studies explored the tunnel water pressure problem and used various solutions (including external water pressure calculation, optimization of tunnel waterproofing and drainage systems, new technologies and materials, etc.) but did not consider how to effectively mitigate the impact of water pressure on the lining structure at the tunnel elevation arch. At the same time, the causes of the increase in water pressure outside the tunnel were analyzed, but none of them considered how to effectively reduce the impact of water pressure at the tunnel elevation arch on the lining structure.

Based on these problems, this paper proposes a new road tunnel drainage concept of three-way drainage. Compared to conventional road tunnel drainage systems, the characteristics of this system improve those of conventional road tunnel drainage systems by setting up a water catchment area (such as the pebble sand pool in Fig. 3) at the elevation arch. After connecting to the central drain through a pipe (with a one-way drain valve inside), excess water is discharged using the pressure difference. This can effectively reduce the high water pressure at the elevation arch while not affecting the surrounding ecological environment by discharging too much water under the action of the one-way valve.

The research in this paper is organized as follows: first, a brief review of the latest advances in highway drainage prevention research is conducted. A detailed design of the depressurization system for the tunnel lining structure of a three-way drainage system is then presented. Finally, a series of numerical simulations are conducted to verify the pressure reduction performance of the three-way drainage system. At the same time, some key influencing factors affecting the three-way drainage pressure reduction are assessed by changing the hydraulic conduction coefficients of the surrounding rock and initial support, the head height, the number of reverse water diversion openings in the back arch, and the secondary lining hydraulic conduction parameters.

## Road tunnels are designed to use drainage

In this section, the research results on drainage prevention in China and abroad are briefly reviewed with the main purpose of highlighting the novelty of the research in this paper.

### Tunnel drainage prevention worldwide

**Figure 1:**
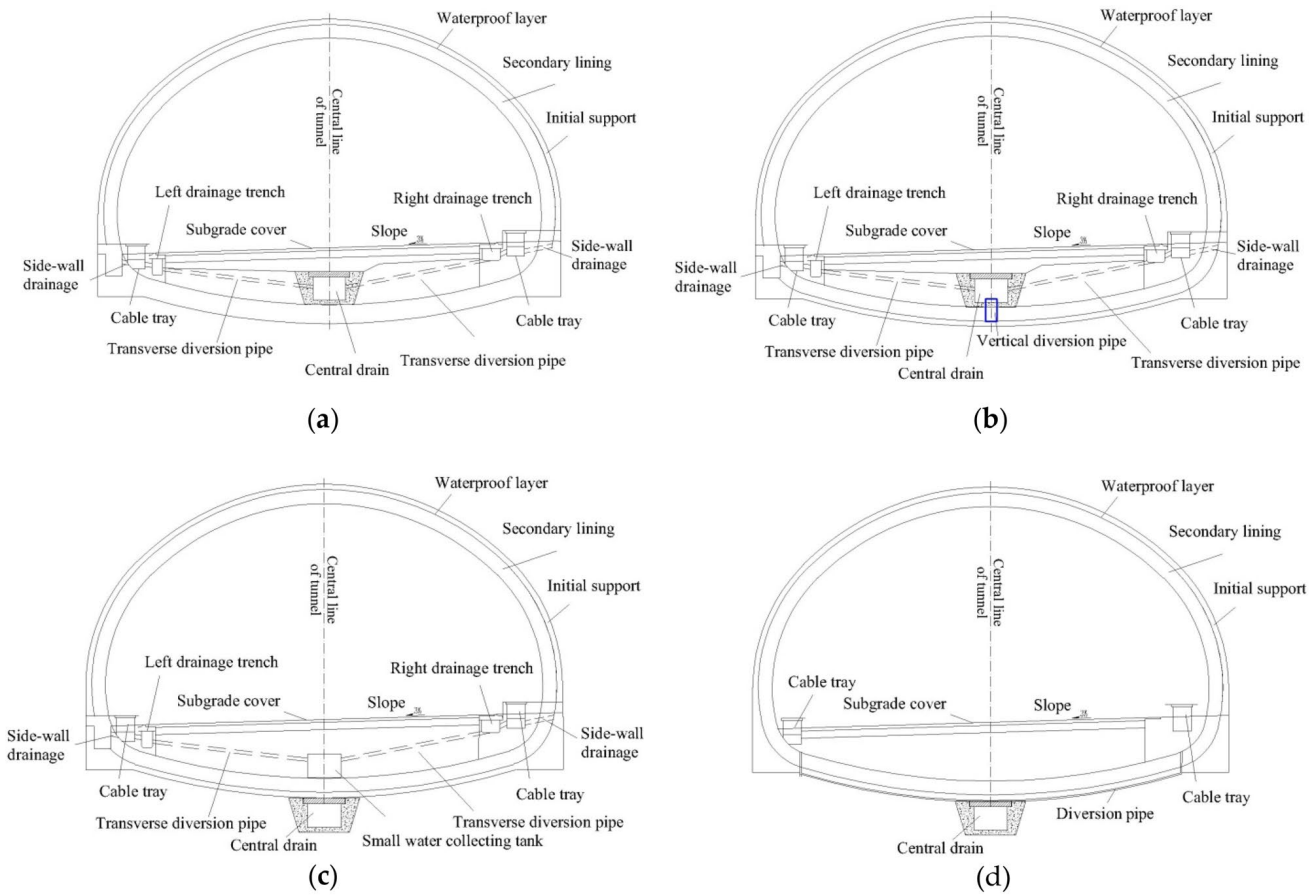
There are four different models that have been studied: (**a**) drainage system A; (**b**) drainage system B; (**c**) drainage system C; and (**d**) drainage system D.

At present, the most advanced tunnel drainage prevention strategies used in China and abroad are derived from Europe, Japan, Sweden, South Korea and other developed regions or countries. In most countries, water-rich mountain tunnels are drained to reduce the external water pressure in the tunnel lining under conditions without special requirements. The structure consists of a flashing between the initial support and the secondary lining, a circular drainage blind pipe, a longitudinal drainage blind pipe, a central drainage trench, and a sinkhole set at 50 m (88 m). As far as the road tunnel drainage system is concerned, the difference lies in the direction of water discharge at the elevation arch and the different pressure-reducing performance of the already studied four models, as shown in Fig. 1^9,30,35^:

1. Circular and longitudinal drainage blind pipes are used to draw out water, and water is introduced into the central drainage trench through transverse diversion pipes. The transverse diversion pipe and the central drainage ditch are installed below the roadbed to form a complete drainage system (Fig. 1a).
2. A central drainage trench is set below the tunnel lining, and the circular drainage blind pipe is directly connected to it for drainage work (Fig. 1b).
3. On the basis of 1, the circular blind pipe is extended below the elevation arch before connecting to the central drain through the reverse drain to form a complete drainage path. A protective gravel head is usually laid around the central drain, which also serves as a filter for debris (Fig. 1c).
4. Therefore, the lining is in complete closure and does not allow water to flow into the tunnel. Outside the body, the water is led out of the tunnel on the basis of 2 in combination with the lateral diversion pipe in 1, which brings the water together in a central drainage trench below the supine arch (Fig. 1d).

### Drainage systems used in Chinese road tunnels

**Figure 2:**
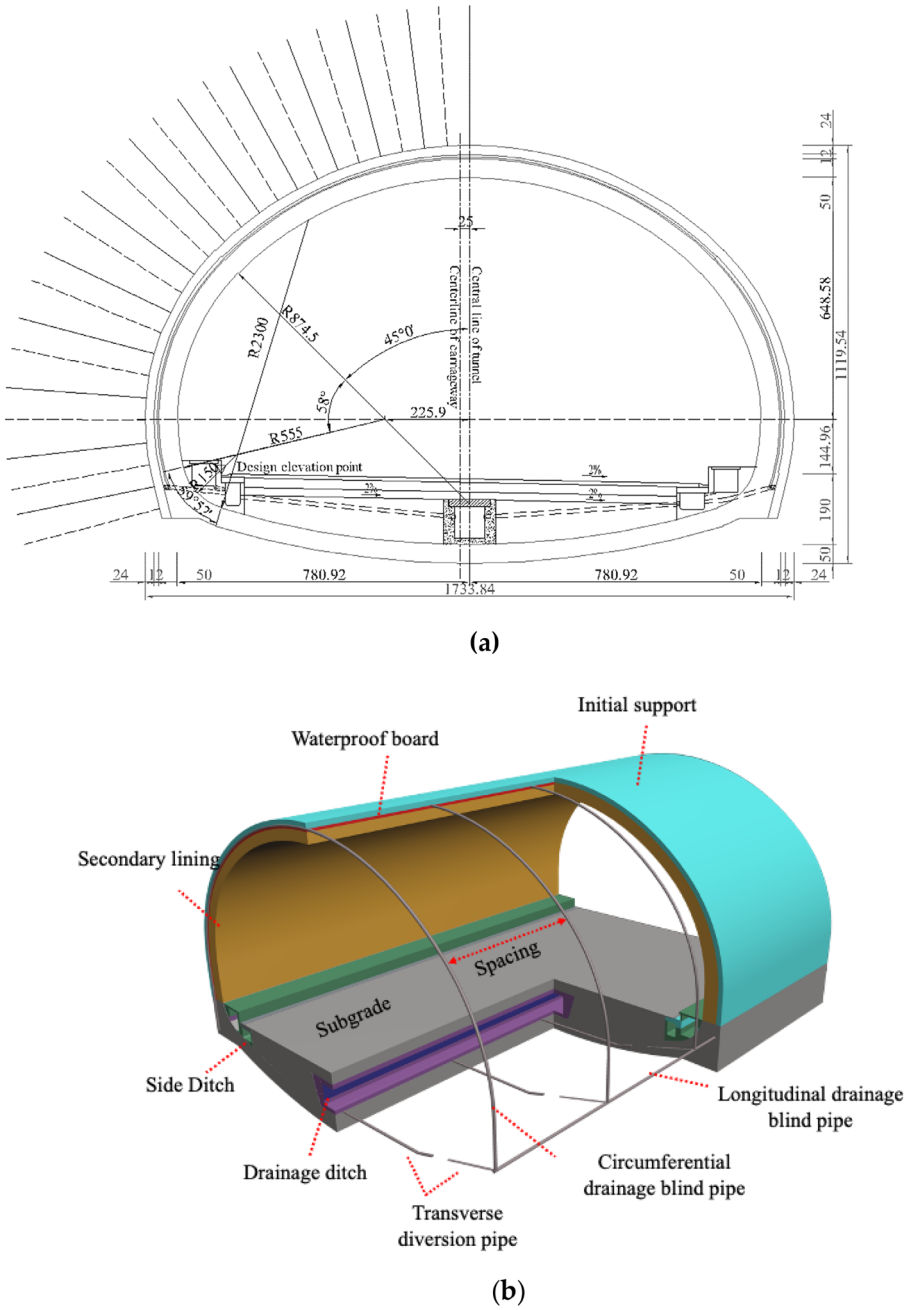
(**a**) Lining structure cross-section size; (**b**) Commonly used anti-drainage systems in Chinese road tunnels.

The current drainage system frequently used in Chinese road tunnels is shown in Fig. 1. The section dimensions are shown in Fig. 2a. Generally, this drainage system is installed between the secondary lining and the initial support with ring and longitudinal drainage blind pipes as well as a waterproof layer, and the central drainage trench and the lateral diversion pipe are installed below the roadbed. The water around the tunnel is directed through the drainage pipes and collected in the central drainage ditch through the lateral drainage pipes. The distance between the drainage pipes in the project and the surrounding rock grade, the amount of water-rich rock and the height of the head have a certain relationship, and the general longitudinal distance between the circular drainage blind pipe is approximately 6 ~ 10 m.

The above illustrates the drainage of the tunnel anti-drainage buck, no matter which drainage is to protect the tunnel safety and service life, but often has certain defects. For example, in Fig. 2, Chinese road tunnels commonly use a drainage system that is easy to implement, but it may be unable to drain the water from the elevation arch position of the tunnel. In the case of no rain, the safety of the tunnel can be ensured, but in the case of relatively high rainfall or long-term operation of the tunnel pipe causing crystallization, the drainage may not be timely, leading to tunnel lining force damage and ultimately affecting the life of the tunnel. As shown in Fig. 1c and d, the central drainage trench is located below the elevation arch of the tunnel. Although it can effectively solve the problem of high water pressure at the elevation arch of the tunnel, it cannot control the amount of groundwater discharge. This will lead to the phenomenon of too much discharge adversely affecting the surrounding ecological environment. It will also have an impact on the construction of the tunnel, resulting in a huge amount of earth and rock excavation, which will not only affect the construction period but also increase the economic investment of the project. In Fig. 1b, although this design adds a reverse drain, no protection is provided for the pipe head. Crystallization or blockage of foreign objects will occur with increased operation, which will lead to a decrease in the drainage volume, thus causing the water pressure at the elevation arch to rise. This is detrimental to the tunnel lining in the long run. To solve the above problems, the following discussion and analysis of the core contents of this paper will be carried out.

### Concept and design of a three-way drainage depressurization system for highway tunnels

This paper studies a three-way drainage and depressurization system for Chinese highway tunnels, which consists of four main components: ① a circular drainage system; ② a longitudinal drainage system; ③ a reverse drainage pipe for the elevated arch; and ④ a transverse diversion pipe and central drainage trench. Among them, the ring, longitudinal drainage blind pipe and the newly added supine arch reverse drainage pipe achieve three directions of drainage purposes in this paper and are called the three-way drainage pressure reduction system (hereinafter referred to as the three-way drainage system). Details of the structure are shown in the figure.

**Figure 3:**
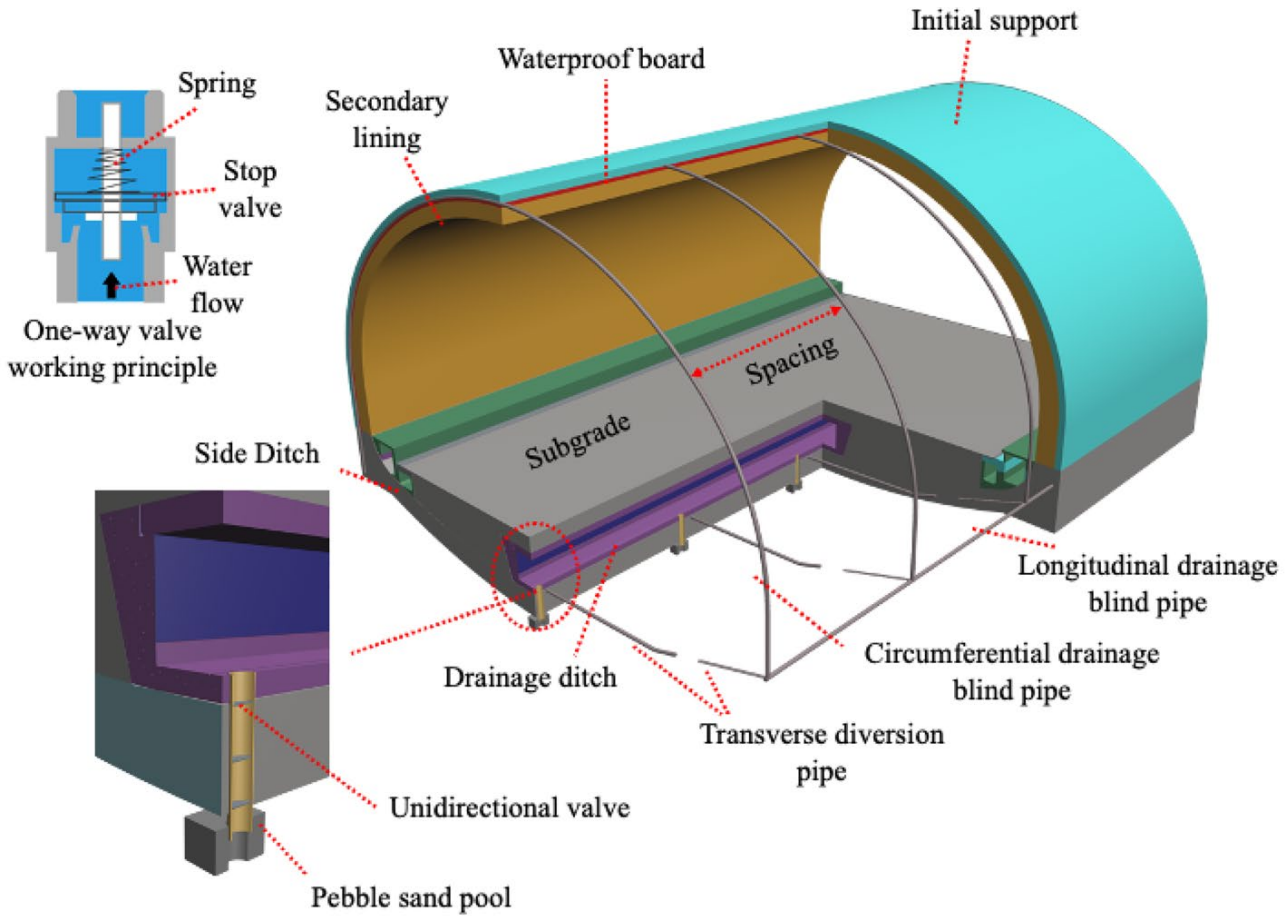
Three-way drainage system structure arrangement.

1. Circumferential drainage system: The circumferential drainage system is located between the initial support and the secondary lining of the tunnel and is responsible for directing the water flow around the tunnel to discharge water, as shown in Fig. 3.
2. Longitudinal drainage system: The longitudinal drainage system is responsible for collecting the water directed down from the circular drainage system as well as the perimeter water seeping down and then bringing the water to the central drainage ditch through the horizontal diversion pipe.
3. Reverse drainage pipe for the supine arch: the reverse drainage system for the supine arch consists of a sand pond (coarse sand and cobble). Outside the tunnel lining, a one-way drainage valve set^36^ (Bgha B, Hui L A et al. 2020) and a reverse drainage pipe are connected to the central drainage ditch, which finally leads to the high water pressure at the supine arch out of the tunnel through the central drainage ditch during the flood season (see Fig. 6).
4. Transverse diversion pipe and central drainage ditch: The horizontal diversion pipe and central drainage ditch are mainly responsible for diverting the water collected by the three-way drainage system out of the tunnel to reduce the water pressure outside the tunnel lining.

**Figure 4:**
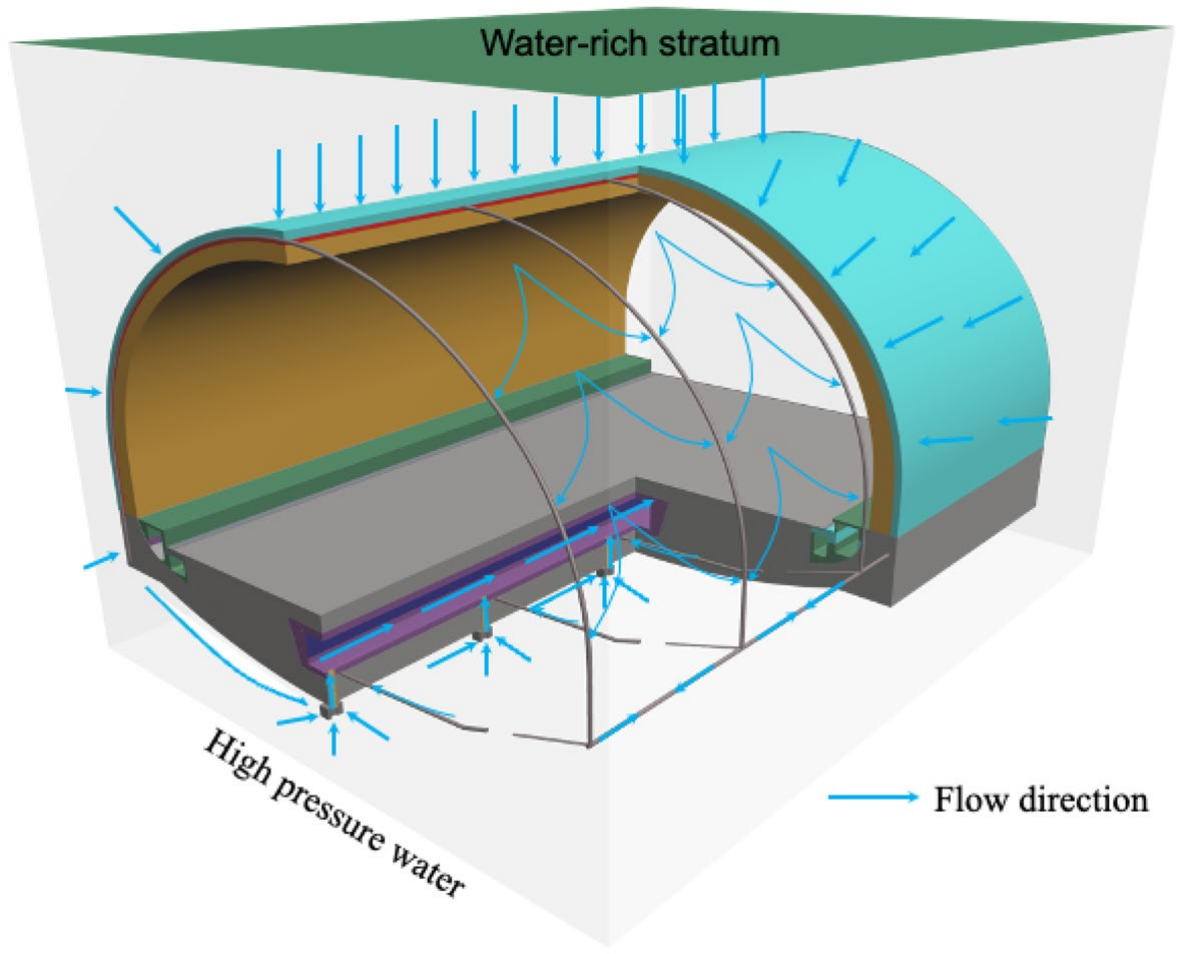
Drainage mechanisms of three-way drainage system.

The structural differences between the conventional drainage system for road tunnels in water-rich areas (Fig. 2) and the new concept drainage system for three-way drainage (Fig. 3) are compared. The so-called three-way drainage system uses the high-water pressure at the elevation arch to divert the high-pressure water out of the tunnel through the collecting sand pond and the reverse drainage pipe to achieve pressure reduction in the elevation arch of the tunnel. The high water pressure at the bottom of the elevated arch in Fig. 4 will drain the water under the pressure difference. The one-way valve prevents water from backing up and drains only when sufficient design pressure is reached at the elevation arch, and the sand pond has a protective effect on the water inlet, which increases the service life. During the dry season, the drainage will be blocked by the drainage check valve so that the groundwater will not be discharged excessively in the water-rich area. To meet the principle of "blocking mainly, limited discharge" of tunnel drainage prevention, sediment backflow can be prevented from blocking the structure. Similar studies are currently available for this concept^30^. Although this study also serves to reduce the high water pressure at the elevation arch, it mainly serves railroad tunnels, and the study structure is relatively complex.

## Numerical simulation analysis

Since this paper proposes a new drainage system, which currently has no case in engineering, numerical simulation is used to study it. In this section, the feasibility of the three-way drainage system concept is investigated by numerical simulation with Midas gtsNX software. The research results demonstrate that the three-way drainage concept can not only reduce the overall water pressure of the tunnel lining structure but also effectively reduce the water pressure at the elevation arch. A detailed analysis will be presented below. The calculation of water pressure outside the liner is mainly based on the theory of well flow in infinite aquifers and Darcy's law.

### Boundary conditions of the numerical models

To study the performance of the three-way drainage system on the water pressure drop at the elevation arch of the tunnel, this paper uses finite element software to simulate the three-way drainage system and the conventional drainage system for comparative analysis and verification.

**Figure 5:**
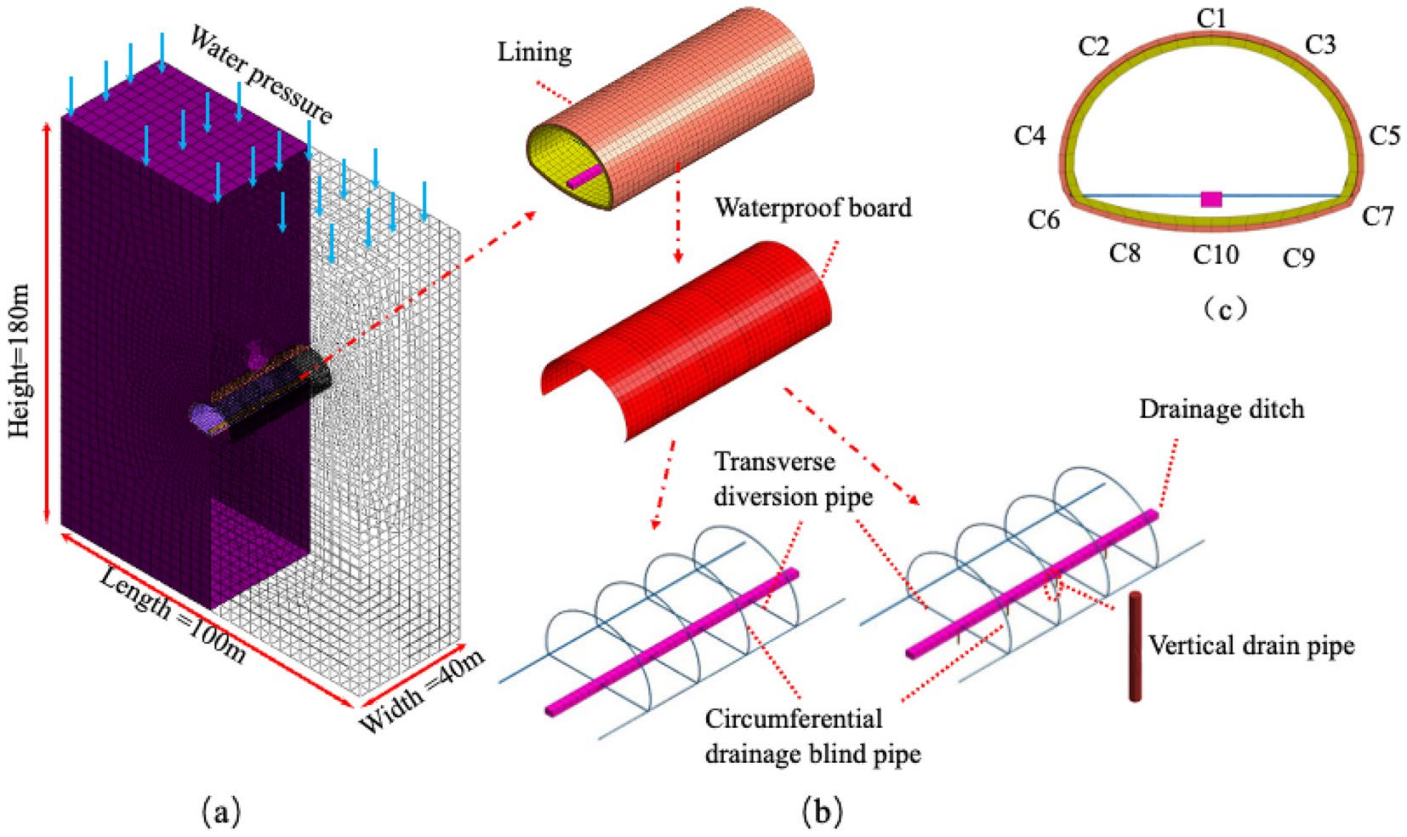
Grid model and drainage structure details and the arrangement of monitoring points: (**a**) Three-dimensional simulation model; (**b**) Conventional and three-way drainage structure; (**c**) Monitoring layout points.

The model simulation is based on the Tongzi Tunnel, a mega-section high-speed tunnel under construction in Guizhou, China, as a reference. The estimated daily water influx in this tunnel reaches 192,281 m^3^/d, with an average precipitation of 1037.3 mm per year, a maximum annual rainfall of 1374 mm and a maximum daily rainfall of 173.3 mm, and the karst depressions in the area are mainly developed in the tunnel inlet and the cave section. The groundwater level at the top of the tunnel vault is 70 m, and the head is applied at the top. The model size of length × width × height = 180 × 40 × 100 m. The perimeter is taken to be greater than 3 by the hole diameter to eliminate the boundary effect, and the mesh model and structure details are shown in Fig. 5. All parts of the model are simulated using solid units. The present model makes the following assumptions: the surrounding rock is simulated using the Mohr‒Coulomb principal structure model, and the initial support, secondary lining structure and drainage prevention system are simulated using the elastic model. The model is always fully saturated, and the nodal head pressure is set to 0 in the drainage hole area to simulate drainage. To facilitate the calculation, the drainage system in an actual project commonly uses a PVC pipe diameter as a reference basis. The modeling is equivalent to a square structure of 89 × 89 × 89 mm using the equal flow principle. The specific parameters of the material are listed in Table 1 below.

**Table 1:** Parameters for each material.

Project	Porosity n	$\gamma$(kN/m^3^)	E (GPa)	$\sigma$	Hydraulic conductivity(m/s)
Secondary lining	0.01	23	31	0.2	1.3 × 10^–11^
IV surrounding rock	0.2	23	5.8	0.12	1.4 × 10^–5^
Initial support	0.12	23.5	28	0.2	1.0 × 10^–7^
Waterproof and drainage layer	0.1	1	1	0.2	1.3 × 10^–9^
A drain	0.5	1	0.65	0.1	0.5

Due to the existence of a one-way valve group in the reverse drainage pipe at the elevation arch, we solve the problem by controlling the hydraulic conduction coefficient, taking a value of 0.3.

### Analysis of results

**Figure 6:**
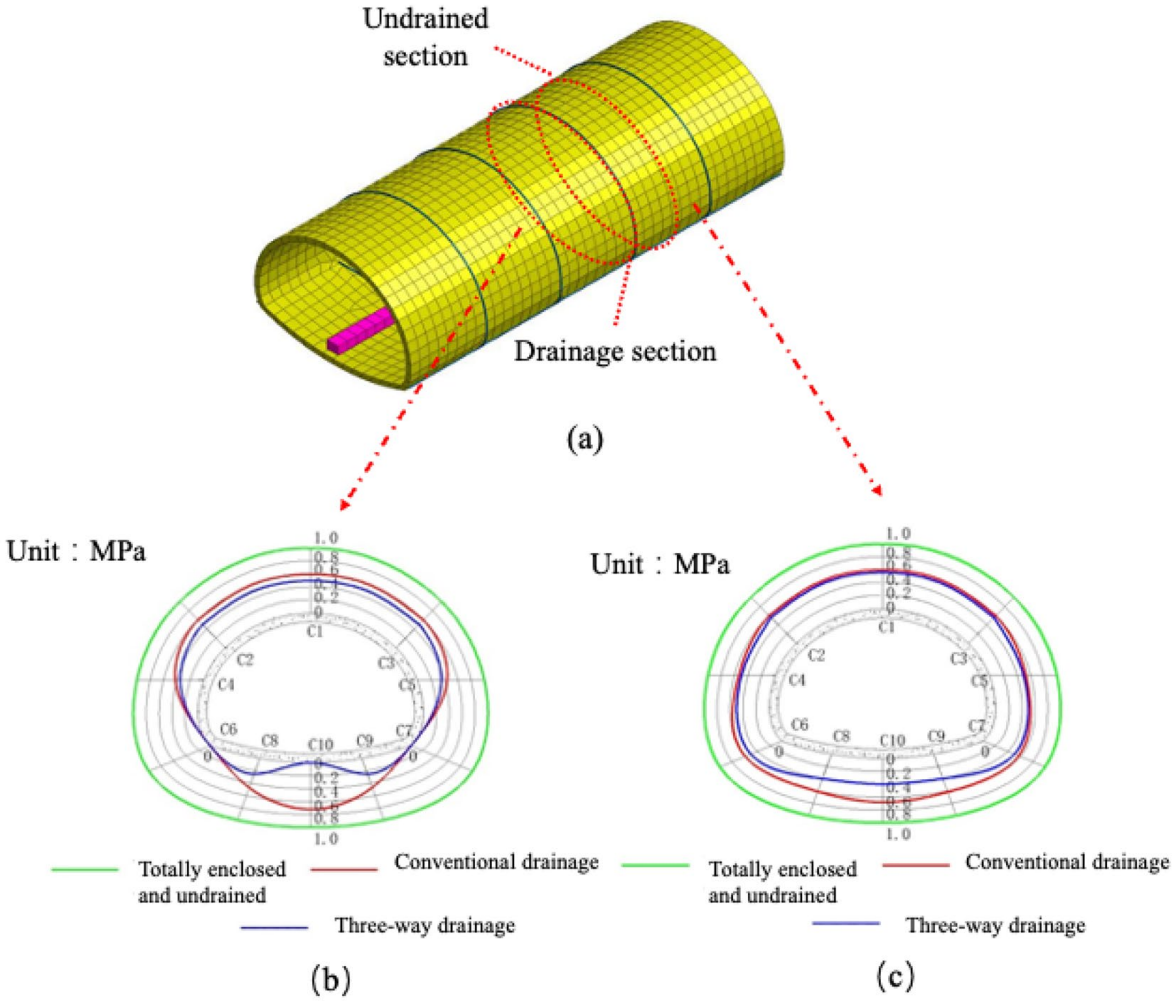
The envelope diagram of external water pressure should be listed as follows: (**a**) Monitoring section; (**b**) water pressure at the drainage section; (**c**) water pressure at the drainage section.

To verify the performance of the three-way drainage system, a comparative analysis of the fully enclosed undrained, conventional drainage system and the three-way drainage system was performed and normalized based on the values of the fully enclosed undrained system. The drainage cross-section and nondrainage cross-section of the tunnel circular monitoring section (C1 to C10) were extracted for analysis. Figure 6a shows water pressure distribution at the drainage section of the secondary lining and (b) water pressure distribution at the nondrainage section of the secondary lining. According to Fig. 6b and c, the following conclusions can be drawn.

Compared to a fully enclosed undrained system with an external water pressure of 1 MPa, a conventional drainage system with an external water pressure of 0.58 MPa effectively reduces the water pressure outside the liner by approximately 58% in the section above the longitudinal drainage pipe. For measurement points C1 to C7, if the drainage system drainage capacity is fully developed, the pressure reduction effect will be more obvious. However, a conventional drainage system does not have a drainage outlet at the bottom of the supine arch. Measurement points C8 to C10 show that the water pressure around the elevation arch is reduced by less than 20%. Therefore, for the time being, the performance of conventional drainage systems in Chinese road tunnels is still lacking in terms of pressure relief at the elevation arch of the tunnel. Such high-water pressure gathered at the elevation arch, if it encounters heavy rain or continuous rainfall, can make the tunnel elevation arch structure highly vulnerable to the threat of high-water level water pressure, thus reducing the life of the tunnel. However, in the study, it was found that the use of reverse drainage of the elevated arch was effective in reducing the occurrence of such situations. The principle is to use the pressure difference to reverse the water out of the tunnel from underneath the elevated arch. The concept of "three-way drainage" in this paper is mainly derived from the concept that pressure difference can cause water to flow in the opposite direction, which can effectively drain the water under natural pressure. This also prevents excessive water discharge, which is beneficial to the surrounding ecological environment.

From the results of numerical simulation (b), the pressure reduction performance of the three-way drainage system at the elevation arch is approximately seven times that of the conventional drainage system, and the external water pressures of the two are 0.09 MPa and 0.68 MPa respectively. The water pressure at the elevated arch is significantly reduced. It is not difficult to find from (c) that there is also a certain effect on the overall pressure reduction of the tunnel structure. The pressure reduction capacity of the nondrainage section is likewise increased, and the pressure reduction effect at the elevated arch is approximately 57% higher than the conventional effect. In the above numerical simulation, the pressure reduction performance and feasibility of the "three-way drainage system" were verified. From the results, it is obvious that the water pressure is greatly reduced after the reverse drainage holes are set in elevation arches C8 and C9.

**Figure 7:**
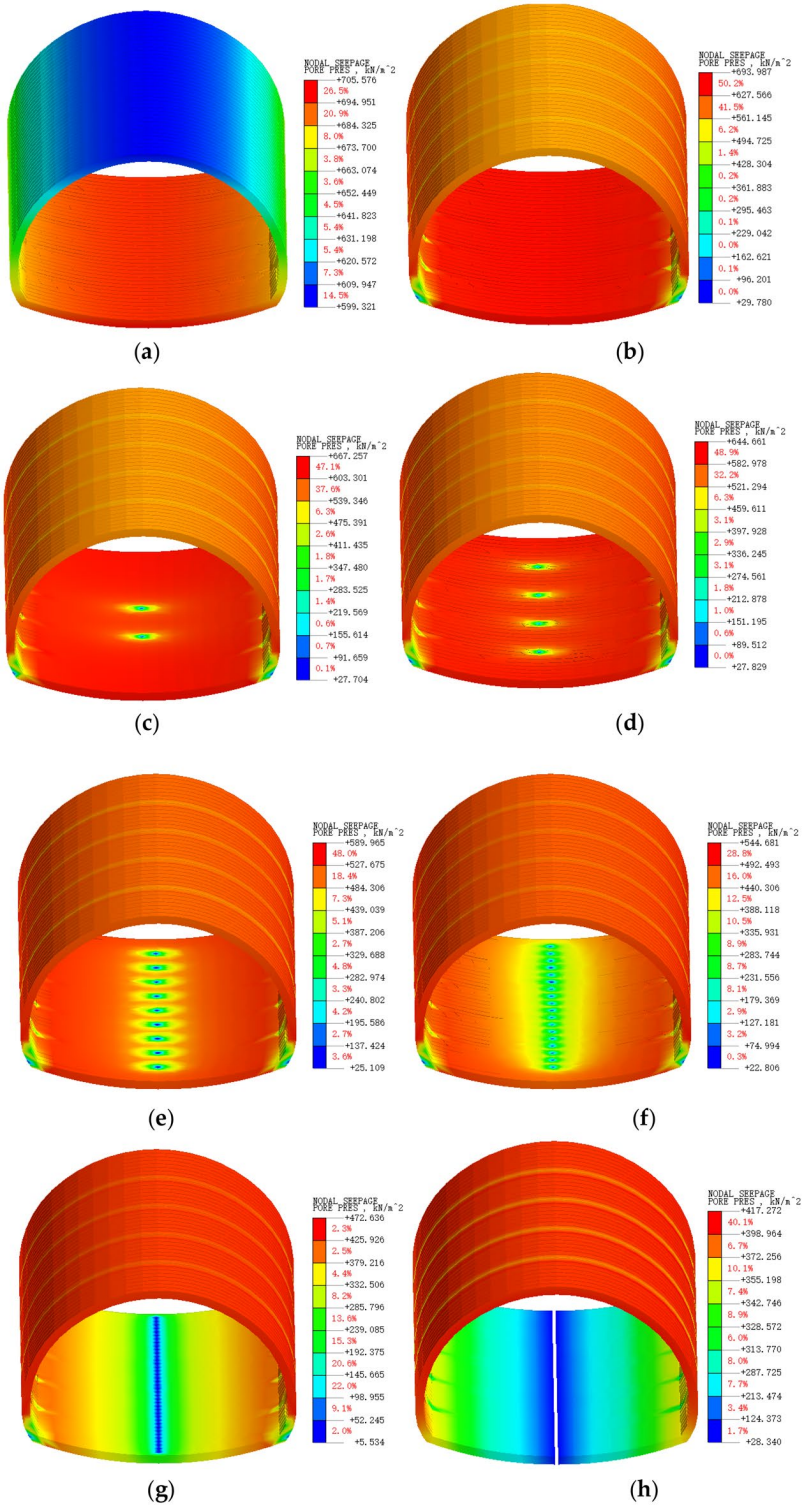
Secondary lining external water pressure cloud: (**a**) No drainage; (**b**) Conventional drainage; (**c**) 2 reverse drains; (**d**) 4 reverse drains; (**e**) 8 reverse drains; (**f**) 16 reverse drains; (**g**) 32 reverse drains; (**h**) Reverse drain.

The following conclusions can be drawn from Fig. 7, where (c) to (h) show the multiplicative relationships of the number of reverse drainage holes in the supine arch for three-way drainage. In the above cloud diagram, it can be seen that the secondary lining in the fully closed state of Fig. 7a is in a state of hydrostatic pressure, and the external water pressure is very high. In Fig. 7b, for the conventional drainage method, the external water pressure is reduced by approximately 30%. However, the elevated arch is still subject to high external water pressure, which is unsafe for long-term operation. For the three-way drainage system, it is obvious that when the number of reverse drainage holes increases from 2 to 32 according to the previous multiplicative relationship, it can be seen that a greater number of reverse drainage holes at the elevation arch is more beneficial for elevation arch pressure reduction. However, for the design of this paper to serve later projects, a reasonable and economical number of reverse drainage holes should be determined. When the number of reverse drainage holes increases from 8 to 16, a decrease of approximately 12% occurs, and the pressure reduction rate is the highest at this time. In Fig. 7h, there is an infinite number of reverse drainage holes, although the pressure reduction effect is significant. However, this is unsafe for the overall structure of the tunnel, so it is recommended to set up 16 reverse drainage holes for every 40 m long interval.

In general, the feasibility of the new drainage concept of three-way drainage was verified. In addition, the reverse drainage of the elevated arch can protect the tunnel in the flood season when the pressure is high, and pressure differences are used so that the water is automatically discharged. Retaining water resources during dry weather does not have a significant impact on the surrounding ecosystem.

## Analysis of factors affecting the performance of the three-way drainage system

In this section, the performance of the three-way drainage system will be studied by numerical simulation parameters varying the parameters of the hydraulic conductivity of the surrounding rock, the head height, the initial support and the secondary lining.

### Parameter assessment and analysis

The following paper presents a numerical simulation study by analyzing the three-way drainage under different parameters. The study of the three-way drainage buck performance was carried out by changing the parameters. The following discussion is carried out under the condition that the drainage is completely usual as well as completely symmetrical. The hydraulic conductivity coefficient and head height of the IV enclosure, secondary lining and initial support are changed under the conditions of Table 1, and the specific parameters are changed as shown in Table 2.

**Table 2:** Parameter values and variations.

Calculation variable	Permeability		Height of water head (m)
	IV surrounding rock	Initial support	
SLH1	0.1, 0.2, …, 1, 2, …, 10		25, 30, …, 70
SLH2			
Number of vertical drains	Conventional drainage	Three-way drainage	
	No vertical diversion pipe	1, 2, …, ∞	

The elevated arch is regarded as the primary support and the second lining superimposed, so the hydraulic conduction coefficient is not considered separately there.

For numerical calculation, first, a variable is selected as a fixed value. Analytical calculations are performed by changing other physical parameters. For example, the hydraulic conductivity of secondary lining 1.3×10^−11^ in Table 1 remains unchanged, and the hydraulic conductivity of other parameters IV surrounding rock and initial support increases 1 to 10 times. Since there are too many combinations, only order changes are considered. The head height increases from 25 m at a rate of 5 m to a head height of 70 m. To see the difference in the result curves, the hydraulic conductivity of the secondary lining takes two fixed values of 1.3×10^−8^ and 1.3×10^−11^ as high and low hydraulic conductivities, respectively, for analysis, which are noted as SLH1 and SLH2 in the following.

The number of three-way drainage pipes continues to increase from 1, 2, …, ∞; that is, the number of reverse drainage pipes of the elevated arch continues to increase from 1 to form a sink, which is ∞. Since it was not possible to model this many times, only 10 values were selected for the simulation. This is because such a value is already highly intensive in this computational model.

### Analysis of parameter values and variation results

The calculations in this section are all in accordance with Table 2 for the analysis of the magnitude values of water pressure affecting the secondary lining outside. The C8 and C9 measurement points with special representation were taken for the results and compared to the fully closed and conventional drainage systems analyzed above. The following plotted results are normalized to the fully enclosed nondrainage results, and after changing one condition, the other conditions are the initial values in Table 1.

#### Hydraulic conductivity of IV surrounding rock

**Figure 8:**
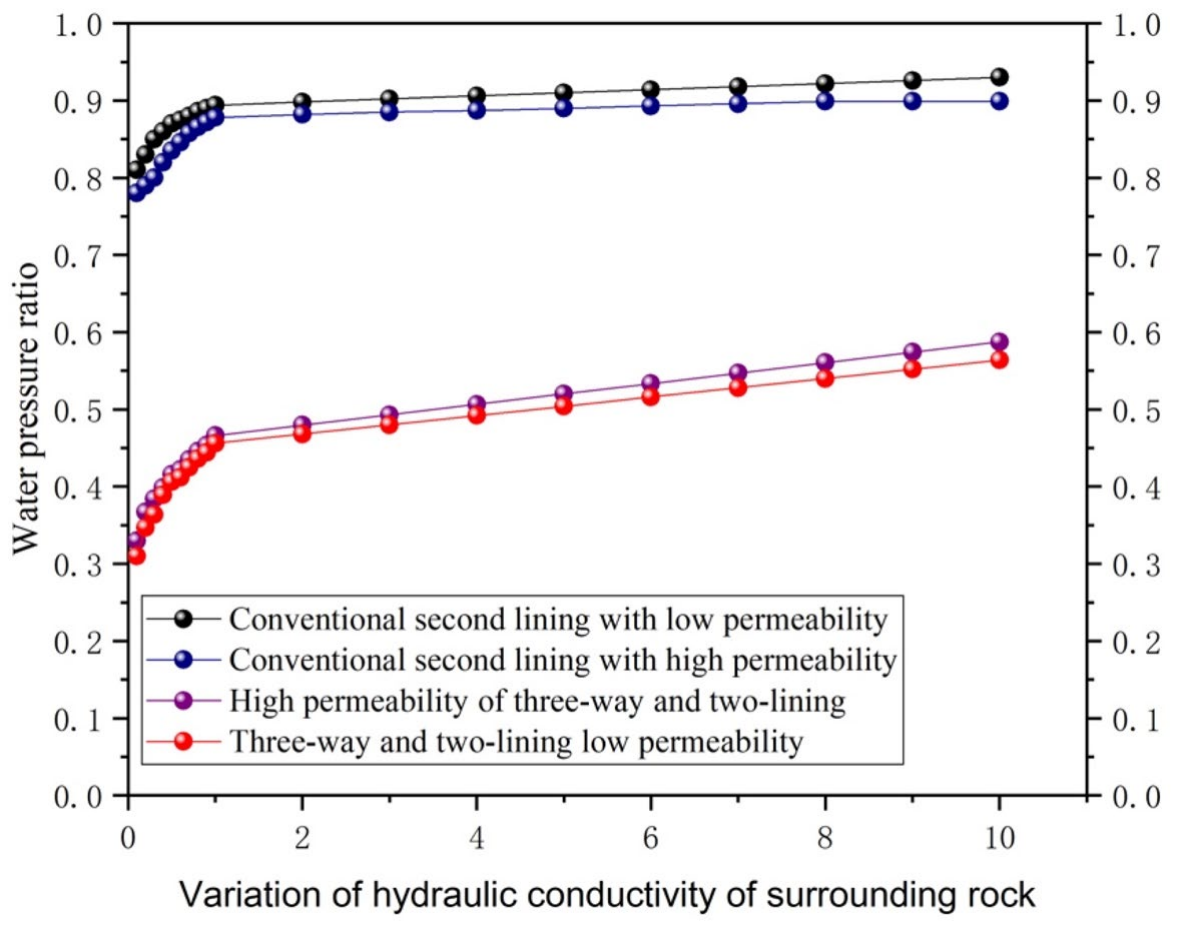
Hydraulic conduction coefficient of IV surrounding rock on the effect of water pressure outside the secondary lining.

The effect of the hydraulic conduction coefficient of the IV surrounding rock on the water pressure outside the secondary lining under the condition of not changing the surrounding rock grade is shown in Fig. 8.

It can be seen in Fig. 8 that the hydraulic conductivity of the IV envelope increases slowly from 0.1, 0.2, ..., 1, 2 times to 10 times when the external water pressure of the secondary lining is increasing. The growth trend is from a rapid increase to steady. The results show that the hydraulic conductivity of the surrounding rock increases the overall external water pressure of the secondary lining, but the high permeability of the secondary lining can effectively reduce the water pressure. Compared with conventional drainage, the three-way drainage elevation arch water pressure in the surrounding rock hydraulic conductivity coefficient in 6 to 10 times the size of the water pressure is maintained at approximately 0.65 times. Therefore, the three-way drainage mode can effectively reduce the external water pressure at the elevation arch of the tunnel. When the hydraulic conductivity of the secondary lining is increased by 100 times, the difference in the water pressure ratio at C8 and C9 is not too great.

#### Hydraulic conductivity of the initial support

**Figure 9:**
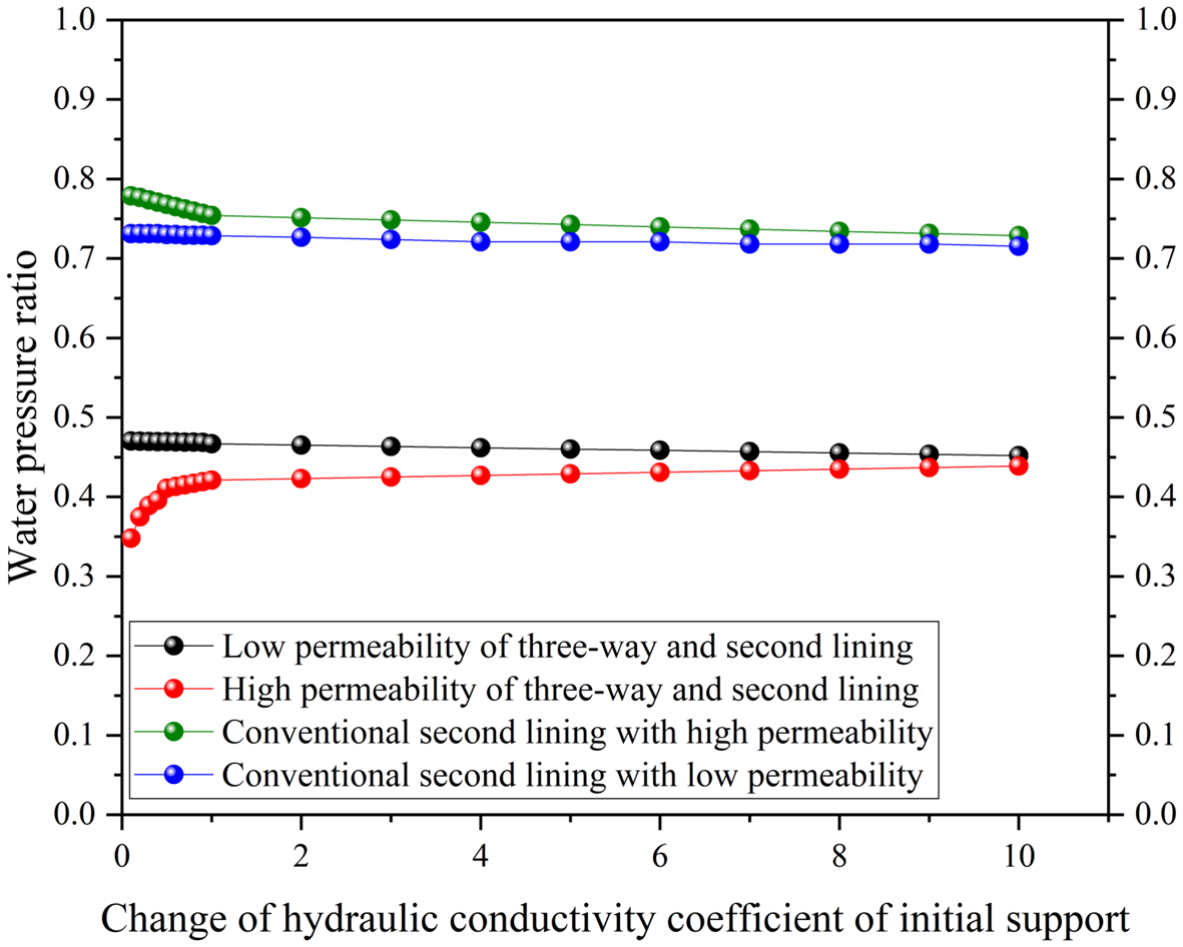
Effect of the hydraulic conduction coefficient of the initial support on the water pressure outside the secondary lining.

Figure 9 shows that the initial support hydraulic conduction coefficient grows, and the water pressure outside the secondary lining of the three-way drainage system grows slowly and eventually stabilizes. For conventional drainage systems, the numerical solution of the water pressure outside the secondary lining slowly decreases and eventually stabilizes. From the results of both, there exists a most unfavorable value for the initial support hydraulic conductivity coefficient. Therefore, the external water pressure of the secondary lining will have a maximum peak. After this maximum value, the water pressure in the secondary lining will tend to decrease, but only within a small range of fluctuations.

#### Increase in the number of reverse drainage holes in the supine arch

**Figure 10:**
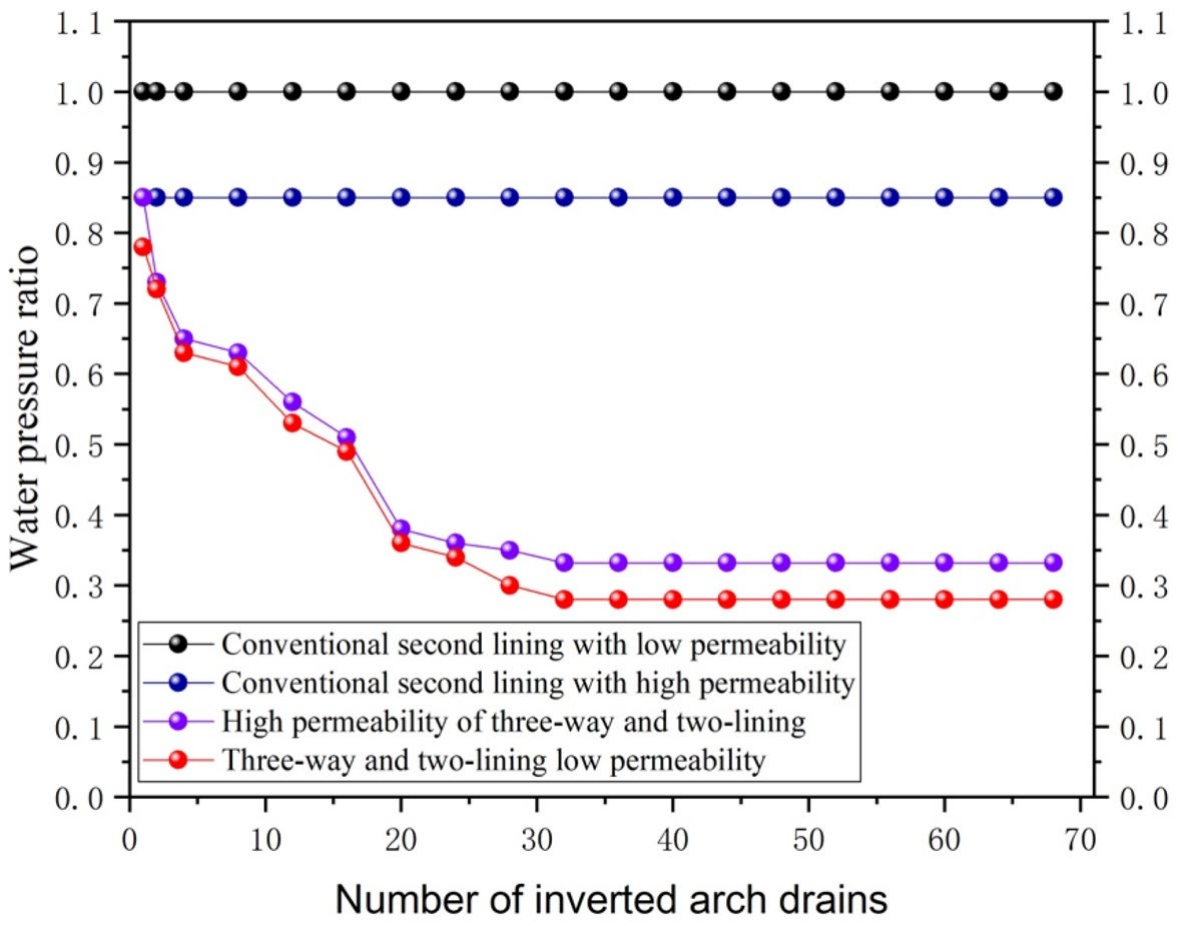
The effect of the number of reverse drainage holes at the elevation arch on the water pressure outside the secondary lining.

As shown in Fig. 10, the drainage capacity is improved by changing the number of reverse drainage holes in the elevated arch. The numerical study found that the external water pressure of the secondary lining decreased regularly with an increase in the reverse drainage hole of the elevated arch. As the number of drainage holes changes from a point to a line, the rate of water pressure reduction becomes increasingly slower, and the curve eventually flattens out. This means that when the number of drainage holes slowly increases to a very dense level, there is no longer a significant increase in the external water pressure of the secondary lining. This shows that there is an optimal number of drainage holes set at the elevation arch for three-way drainage. For example, in this paper, if the lining length is 40 m, the number of 16 reverse drainage holes of the elevated arch will be set to reach the optimal value. At the same time, the three-way drainage method can also effectively reduce the overall external water pressure of the elevated arch and secondary lining compared with conventional drainage.

#### Water head height variation

**Figure 11:**
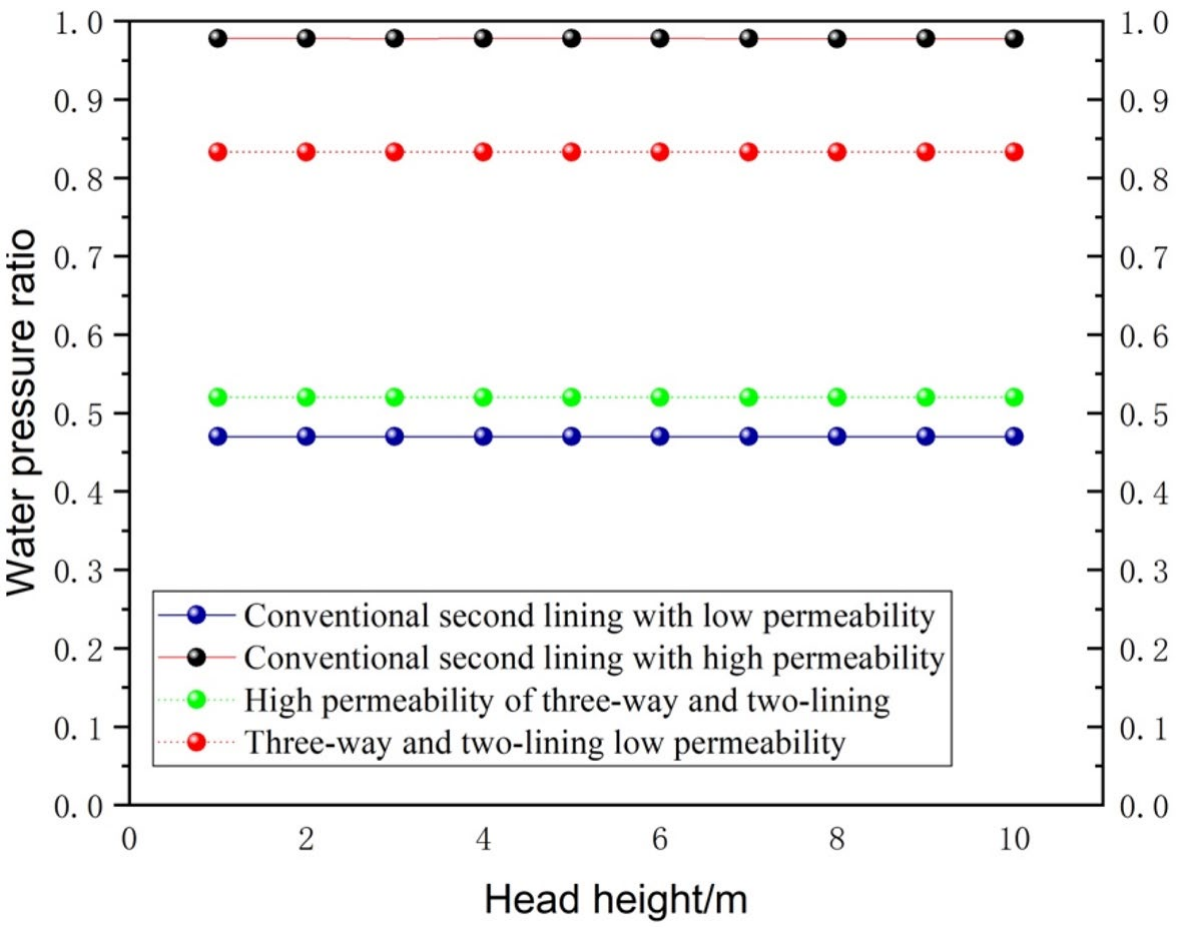
Different head heights on the secondary lining outside the water pressure effect.

Other initial conditions remain unchanged, and the head height change on the secondary lining outside the water pressure influence law is shown in Fig. 11. It is not difficult to find that with the increase in head height, the external water pressure of the secondary lining is basically unchanged. This indicates that as the head increases and the water pressure increases, the size of the discharged water flow will also increase year-on-year. Since only the highest head of 70 m is analyzed here, this is different from the case of deeply buried tunnels. Therefore, the increase in head height has little effect on the external water pressure of the secondary lining for lots where the burial depth is not large. However, compared with conventional drainage, the water pressure reduction after setting the reverse diversion inlet of the elevated arch for three-way drainage is significant, only 0.43 times that of conventional drainage. With different hydraulic conductivity coefficients for the secondary lining, it is not difficult to find that the difference between the two is not large under the 100 times multiplier condition, indicating that this is not a major factor.

## Conclusion

To efficiently and economically solve the damage problems caused by high water pressure in the elevation arches of highway tunnels in China, this paper proposes a new drainage concept of "three-way drainage", which has been found to be effective in reducing the high water pressure at the elevation arch of the tunnel. The drainage and decompression characteristics of the three-way drainage system on the tunnel lining are analyzed in a numerical simulation. The main findings are as follows:

1. The three-way drainage system adds a reverse drainage structure (with a one-way valve set) to the elevated arch, which is theoretically feasible. It can effectively reduce the external water pressure at the elevation arch of the tunnel as well as the water pressure of the overall structure of the lining.
2. The external water pressure of the tunnel secondary lining has a certain relationship with the hydraulic conductivity of the surrounding rock, which shows a synchronous growth trend.
3. For the numerical results of the three-way drainage in this paper, the increase in the hydraulic conductivity of the initial support has little effect on the change in water pressure in the secondary lining. The change in the force transfer coefficient will cause a peak in the external water pressure of the secondary lining, causing the secondary lining water pressure to begin to level off.
4. The increase in head height makes the water pressure ratio outside the secondary liner increase linearly. Therefore, in an environment of high head height, the tunnel drainage prevention system as well as the lining structure will need to improve the design requirements.

The three-way drainage system in this paper has demonstrated as feasible. It is not currently used in practice and is still at the stage of theoretical analysis. Indoor model tests will be conducted to provide a reasonable and economic drainage method for road tunnels, and actual engineering experience will be used to verify the drainage and pressure reduction performance of the three-way drainage system.

## Data Availability

The datasets used and/or analyzed during the current study are available from the corresponding author on reasonable request.
